# Impact of *in vitro* diagnostic tests in health, society and economy in Portugal

**DOI:** 10.3389/fpubh.2026.1715491

**Published:** 2026-02-20

**Authors:** Paula Rodrigues, Sílvia Mota, Carolina Amaral, Bruno Garganta, Carlos Catalão, Liliana de Almeida, Inês Teixeira, Paula Costa, Paulo Dias

**Affiliations:** 1 Antares Consulting, Lisbon, Portugal; 2 Werfen, Lisbon, Portugal; 3 Roche Sistemas de Diagnósticos, Lisbon, Portugal; 4 Portuguese Pharmaceutical Industry Association – APIFARMA, Lisbon, Portugal; 5 Comprehensive Health Research Centre, Universidade de Évora, Évora, Portugal

**Keywords:** COVID-19, diabetes, heart failure, *in vitro* diagnostic (IVD) test, lung cancer, social return on investment (SROI)

## Abstract

**Introduction:**

*In Vitro* Diagnostic (IVD) tests play a fundamental role across the healthcare continuum by enabling early detection, guiding treatment decisions, and supporting disease monitoring. Despite representing a small fraction of health expenditure, IVD tests generate substantial clinical, social and economic value. This study aims to generate evidence on the contribution of IVD tests to health, society and economy, considering its impact at different stages of diagnosis or follow-up, in four pathologies with high or increasing incidence or prevalence, and public health relevance in 2021: COVID-19, diabetes, heart failure, and lung cancer.

**Methods:**

A Social Return on Investment (SROI) methodology was applied to evaluate the social and economic return obtained for each euro invested in IVD tests over a 1-year period, across the selected conditions. The analysis was supported by scientific articles, official data, grey sources, and perspectives gathered from stakeholders through interviews and focus groups. For each pathology, an impact map was developed to identify inputs, outputs and outcomes from the perspective of patients, family/caregivers, healthcare professionals, healthcare providers, healthcare systems and society. A consistently conservative approach was adopted in estimating quantities and monetary valuations.

**Results:**

The estimated SROI ratios revealed substantial returns: €8.2 for every €1 invested in PCR tests and professional Ag-RDTs for COVID-19, highlighting their importance in the diagnosis and transmission control; €6 for every €1 invested in glycated hemoglobin and glucose self-monitoring tests for diabetes; €12.6 for every €1 invested in B-type natriuretic peptide tests after prescription in primary care; and €14.9 for every €1 invested in molecular testing, specifically next-generation sequencing, in stage IV lung adenocarcinoma. These returns reflect improvements in health outcomes, healthcare efficiency, and productivity.

**Discussion:**

The case studies revealed economic and social returns ranging from six to fifteen times the initial investment. The findings demonstrated that investing in IVD tests improves clinical decision-making, enhances quality of life, and reduces healthcare costs. Policymakers should recognize IVD tests not merely as diagnostic tools, but as strategic levers to improve public health and economic resilience. These four case studies demonstrate IVD tests value and provide a snapshot of the wider benefits of its applications.

## 1 Introduction

*In Vitro* Diagnostic (IVD) tests play a critical role in modern healthcare, serving as fundamental tools across the continuum of care—from disease prediction and prevention to diagnosis, prognosis, personalized treatment, and ongoing monitoring (1, 2). Performed on human biological samples in either laboratory settings or near-patient contexts, such as Point-of-care (PoC), IVD tests rank among the most frequently prescribed diagnostic procedures, often surpassing diagnostic imaging in clinical workflows (3).

Growing evidence indicates that information from IVD tests influences up to 70% of clinical decisions, directly impacting patient outcomes and contributing to healthcare system efficiency and sustainability. The first reference dates to 1996, where Forsman (4) stated that “laboratory tests represent only 5% of the hospital budget, yet they contribute to around 60–70% of all clinical decisions.” Years later, Rohr et al. (5) conducted a research study to evaluate the impact of IVD tests in health expenditure in two developed markets (the USA and Germany) and to assess their value in clinical decision-making. Their findings concluded that around 66% of clinical decisions are based on IVD tests results.

Beyond their clinical utility and direct contribution to diagnosis and treatment selection, IVD tests generate significant value across multiple dimensions, and for a wide range of stakeholders, including patients, families, healthcare professionals, providers, healthcare systems, and society at large (6). In this context, Wurcel et al. (6) introduced the value of diagnostic information (VODI), an holistic framework that “goes beyond conventional cost-effectiveness metrics by including the “value of knowing” as well as quality of life improvements arising from this knowledge gain.” In their paper, the authors outline the multidimensional value of diagnostic information, which contributes to resource optimization, reducing errors, improving care pathways, and enhancing societal productivity (6).

However, assessing the value of diagnostic tests poses specific methodological challenges. Unlike pharmaceutical and medical interventions, diagnostic tests rarely improve health outcomes directly. Instead, their impact is mediated through downstream clinical decisions and subsequent interventions along the test-treatment pathway (7). As highlighted in the health technology assessment (HTA) literature, traditional evaluation approaches, largely developed for interventions with direct clinical effects, often struggle to adequately reflect the indirect and system-wide contributions of diagnostic technologies (7, 8). As a result, the value of diagnostics is frequently underestimated in decision-making processes.

Building on the concept of VODI, there is growing recognition that the contribution of diagnostic tests extends beyond health outcomes typically captured in cost-effectiveness analyses. Diagnostic information generates value not only by supporting more accurate and timely clinical decisions, but also by reducing uncertainty, improving patient and clinician confidence, optimizing care pathways, and strengthening health system performance. Many of these benefits are particularly relevant from a public health and system perspective yet remain insufficiently captured by conventional HTA frameworks. In this context, the Social Return on Investment (SROI) methodology offers a complementary approach to assessing the broader value of diagnostic testing. By adopting a stakeholder-centered and system-wide perspective, SROI enables the capture of social, economic and organizational outcomes generated along the test-treatment pathway, extending beyond the objectives of traditional cost-effectiveness analyses.

Despite these limitations in value assessment, the available literature consistently shows that the representativeness of IVD tests in health expenditure is relatively small, especially when compared to the value they provide to both user and the health system as a whole (5, 9, 10). In Portugal, for example, the IVD market was valued at approximately €303 million in 2020 (11), while total health expenditure reached €21,108 million euros in the same year (12). This implies that IVD tests accounted for only about 1.4% of total health expenditure, reinforcing the notion that diagnostics represent a modest cost component with potentially disproportionate impact on health outcomes and system performance (2, 13).

Against this background, this article aims to contribute to the growing literature on the economic and social value of diagnostics by applying the SROI methodology to four IVD case studies within the Portuguese healthcare context. Focusing on four high-incidence and high-prevalence diseases with significant public health implications in 2021 (14): COVID-19, diabetes, heart failure (HF), and lung cancer, the analysis seeks to estimate the value created for multiple stakeholders per euro invested in specific IVD applications. By adopting a system-wide and stakeholder-oriented perspective, this study provides new insights into the contribution of IVD tests in public health, health system performance, and societal well-being and economy.

## 2 Materials and methods

### 2.1 Study design

A mixed-method, multi-case design to evaluate the economic and social value of IVD tests in Portugal, using the Social Return on Investment (SROI) methodology. The analysis focused on specific IVD tests applied to four different clinical contexts: (i) PCR and professional antigen rapid diagnostic tests (Ag-RDTs) for COVID-19; (ii) Glycated hemoglobin (HbA1c) and self-monitoring of blood glucose (SMBG) for diabetes; (iii) B-type natriuretic peptide testing for early diagnosis of heart failure (HF) in primary care; and (iv) Next generation Sequencing (NGS) for stage IV lung adenocarcinoma (Table 1). The SROI approach was applied separately to each case.

**Table 1:** Case studies overview.

Case	IVD test	Population	Year of analysis
COVID-19	PCR and professional Ag-RDT	General population tested	July 2020 to June 2021
Diabetes	HbA1c and SMBG	People with type I and II diabetes	2019
Heart Failure	B-type natriuretic peptide test	Adults ≥ 25 years with suspected HF in primary care	2019
Lung Cancer	NGS	Adults diagnosed with stage IV adenocarcinoma	2019

### 2.2 SROI framework

SROI is a comprehensive impact assessment methodology that quantifies the value created for multiple stakeholders by converting social outcomes into monetary terms. It calculates the ratio between the total social value generated and the investment required to achieve that value. The analysis followed the six standard stages of SROI, as outlined by Social Value UK and other international guidelines (15):

- Establishing scope and identifying key stakeholders: for each case study, a clear scope and timeframe were defined (typically 1 year, 2019 or 2020 depending on the case). Key stakeholders were mapped, including patients, families and/or caregivers, healthcare professionals, providers, healthcare system, and society.
- Developing an impact map: the direct results (or outputs) of the analyzed activities were determined. Also, using the “theory of change,” the likely outcomes for each stakeholder group were identified, based on clinical evidence, stakeholder consultation, literature, and expert opinion.
- Evidencing outcomes and assigning a value: quantitative and qualitative data were collected or modeled for each outcome. Each outcome was assigned an indicator and a financial proxy based on different approaches, including: willingness-to-pay (reflecting the value of an outcome according to how much stakeholders are willing to pay or accept for it), economic valuation (representing actual costs or savings incurred by stakeholders), and replacement value (based on the cost of alternative goods or services that would produce an equivalent effect). Financial proxies were derived from national databases, published economic valuations (e.g., QALY valuations, average productivity losses), or market prices where applicable.
- Establishing impact: to avoid overestimation, adjustments were applied to each identified outcome, like deadweight (which corresponds to the percentage of the outcome that would have occurred regardless of the intervention of the activity under analysis) and attribution (which represents the percentage of the outcome attributable to the intervention of other individuals and/or institutions).
- Calculating the SROI: the total adjusted value of the outcomes was summed (total impact) and divided by the total investment (or input) to generate a SROI ratio for each case (Eq. (1)), which represents the amount of social value created per euro invested:

$$\text{SROI}=\frac{\text{Total Impact}\;(\text{€})}{\text{Total Investment}\;(\text{€})}$$

To assess the robustness of the results and address uncertainty, sensitivity analyses were conducted on the most influential parameters in each case study. These parameters were varied by ±20%, and the resulting effect on the SROI ratio was assessed.

vi. Reporting, using and embedding: assumptions, sources, and uncertainties were explicitly documented. A conservative approach was applied throughout the analysis.

### 2.3 Data sources

The SROI analyses were supported by scientific articles, official data, grey literature, stakeholder interviews and focus groups. Each case study used a combination of data sources, including: National epidemiological data [e.g., Our World in Data (16), DGS Reports (17, 18), National Oncology Registry (19)]; Published literature and meta-analyses, including Portuguese health economic studies [e.g., Pinto et al. (20), and Fonseca et al. (21)]; National and International Guidelines and Recommendations [e.g., Teixeira et al. (22), and ESC (23)]; Official price lists [e.g., Ordinance no. 254/2018 (24), and Table of “Publicly Reimbursed Parameters for Primary Care 2021” (25)].

Outcomes were assessed and monetized using a SROI value map developed in Microsoft Excel, adapted from the official Social Value International Value Map available online (26).

### 2.4 Ethical considerations

As part of the SROI analysis, qualitative data was collected through 15 semi-structured interviews and one focus group involving 7 participants to validate assumptions, outcomes, and stakeholder perspectives across the four case studies. Participants included health professionals, patients, patient representatives, support associations, and entities responsible for conducting IVD tests, with relevant expertise to each diagnostic context.

All participants were invited via institutional channels and provided informed verbal consent prior to their participation. Anonymity and confidentiality were ensured, with no personal identifiable information recorded or used in the analysis. Given the non-interventional and voluntary nature of the engagement – without the collection of health or identifying data – formal ethical approval was deemed unnecessary.

## 3 Results

The results of the analysis are presented according to the different phases of the SROI methodology, organized by case study.

### 3.1 Case study: the use of IVD tests in the COVID-19 pandemic

This case study assessed the social impact of using professional PCR and professional Ag-RDTs for the diagnosis and containment of COVID-19 in Portugal, between July 2020 and June 2021. The analysis adopted a comparative scenario in which these tests had not been implemented, quantifying the social and economic impacts prevented or mitigated through early diagnosis and transmission chain control.

#### 3.1.1 Scope and stakeholders

The analysis focused on a total of 12,136,368 PCR and professional Ag-RDTs conducted across public and private institutions, in Portugal (16). The key stakeholders included patients and families as primary beneficiaries of reduced transmission and informed self-isolation, healthcare system and providers, who benefited from reduced pressure and more efficient resource allocation, healthcare professionals, whose safety and workflow continuity were supported through testing, and society at large, which experienced improved productivity and reduced disruption.

#### 3.1.2 Mapping outcomes of PCR and professional Ag-RDTs use

Between July 2020 and June 2021, a total of 12,136,368 PCR and professional Ag-RDTs were conducted across public and private laboratories and testing centers in Portugal. From these, 837,416 positive cases were identified (16). Considering that some cases may have required confirmatory testing, a conservative assumption was applied: each positive case corresponds to two tests, leading to an estimated 10,461,563 negative test results. Based on national epidemiological modeling and observed transmissibility dynamics during the period (27), it was estimated that each confirmed case prevented at least one additional infection, through timely isolation and contact tracing. Consequently, the total number of COVID-19 cases prevented is estimated at 837,416.

These avoided cases translated into eight significant health, social and economic outcomes, including improved quality of life (due to serious sequelae and deaths avoided), enhanced social and psychological well-being, better physical health, increased perceived safety among test users and health professionals, and improved continuity of work and healthcare service delivery. Of these outcomes, four are attributed to users and their families, two to the health system and providers, one to health professionals and two to society. All outcomes derived from the expected chain of events triggered by timely diagnosis and isolation of COVID-19 cases are reported by stakeholder group in Table 2.

**Table 2:** COVID-19 impact map: quantity of change for outcomes by stakeholders.

Outcomes	Indicator	Number	Financial proxy	Adjustment	Impact calculation (€M)
Patients and families					
Improved quality of life due to serious sequelae and avoided deaths	QALYs gained through avoided morbidity and mortality (36)	68,599	WTP per capita [2x national GDP per capita (37)] *€ 41,694.00*	20% dwt 25% atb	1,716.0
Improved physical well-being	Number of mild symptomatic COVID-19 cases avoided	394,814	Physical Activity (Monthly gym fee) *€ 36.74*	20% dwt 25% atb	8.7
Improved social well-being	No. of asymptomatic and mild symptomatic COVID-19 cases avoided	813,522	Average monthly spending per adult on leisure activities (38) *(€ 210.08)*	20% dwt 25% atb	102.5
	No. of negative COVID-19 tests providing social well-being due to infection exclusion	10,461,536	Average biweekly spending per adult on leisure activities (38) *€ 105.04*	20% dwt 10% atb	791.2
Increased sense of security	No. of COVID-19 cases identified who adopted measures to avoid further infections	837,416	Psychological Support (1 session per test) *€ 60.00*	20% dwt 10% atb	36.2
	No. of negative COVID-19 tests that provided tested individuals a sense of safety due to infection exclusion	10,461,536			451.9
Healthcare system and providers					
Reduced hospitalization burden	No. of hospitalization days (including ICU) avoided	600,164	Average daily cost of COVID-19 hospitalization (39) *(€ 643.86)*	20% dwt 25% atb	231.9
Reduced Burden of Long COVID Care	No. of avoided medical appointments due to reduction in Long COVID	167,483	NHS follow-up medical appointment cost (40) *€ 31.00*	20% dwt 25% atb	3.1
Healthcare professional					
Improved workplace safety and well-being	No. of tests (positive or negative) performed on healthcare workers	1,495,224	Psychological Support (1 session *per* test) *€ 60.00*	20% dwt 10% atb	64.6
Society					
Increased productivity	No. of work absence days avoided due to reduced hospitalizations in the working-age population	347,180	Average daily cost per worker (41) *€ 59.20*	20% dwt 25% atb	12.3
	No. of work absence days avoided due to reduced isolation in the working-age population	2,322,317			82.5
Economic improvement	Estimated number of general lockdown months avoided	3	Difference in average GDP, lockdown (Mar–Apr 2020) vs. post-lockdown (Jul 2020–Jun 2021) (42) € 947,741,666.67	20% dwt 10% atb	2,047.1
Total					5,548.2

dwt, deadweight; atb, attribution.

#### 3.1.3 Evidencing outcomes and assigning value

The quantified outcomes in this case study were supported by national epidemiological data, published literature, and widely accepted economic valuations. For instance, the outcome related to improved quality of life—measured in QALYs gained through the prevention of morbidity and mortality (n = 68,599 QALYs)—was monetized using the societal willingness-to-pay (WTP) threshold, set at twice the national GDP per capita in 2021 (€41,694.00). This threshold falls within the cost-effectiveness ranges of €10,000 to €100,000 per QALY defined in the methodological guidelines for economic evaluation studies by Infarmed, even though this is not an economic evaluation (28).

From the perspective of the health system and healthcare providers, the reduction in hospitalization burden was estimated based on the number of hospitalization days avoided (*n* = 600,164) and monetized using the average daily cost of COVID-19 hospitalization (€643.86). The productivity-related outcome was calculated by assessing the number of work absenteeism days avoided, both from reduced hospitalization (*n* = 347,180 days) and isolation periods (*n* = 2,322,317 days) among the working-age population. This was monetized using the average daily productivity cost per worker (€59.20).

A comprehensive breakdown of each outcome, including corresponding indicators and financial proxies, is provided in Table 2. Further details on data sources and underlying assumptions are available in Supplementary Table S1.

#### 3.1.4 Establishing impact

To assess the benefits attributable to the activity under analysis, adjustments were made for deadweight, assuming that some infection reduction would have occurred through spontaneous behavioral change, and for attribution, considering that isolation and public health messaging contributed alongside testing (Table 2). Details about the adjustments are presented in Supplementary Table S2.

#### 3.1.5 Calculating the SROI

The total investment was estimated at € 680.6 million, representing the value of IVD tests carried out in Portugal, during the study period (Table 3). The total estimated social impact, considering only quantifiable outcomes and after the adjustments, reached €5,550 million. The improvement in the economy contributed to the largest proportion of the social impact (€ 2.05 billion or 37% of total social value) followed by the enhancement in individuals’ quality of life due to serious sequelae and deaths avoided (€ 1.72 billion; 31%). Improved physical well-being and reduced burden of care for Long COVID patients contributed the least to the overall impact (Table 2).

**Table 3:** COVID-19: estimated inputs allocated to PCR and professional Ag-RDTs use.

Stakeholders	Resources	Value (€M)	Rationale
Patients and families	Time	–	The time spent undergoing PCR and professional use Ag-RDT was deducted from the benefit associated with avoided absenteeism due to prevented isolation. The cost of testing borne by patients and families is included under the healthcare system.
Healthcare system and care providers	Human resources, PCR and Ag-RDTs	680.6	Investment in PCR tests: Number of PCR tests conducted during the period, assuming they represented around 79.3% of the total tests performed in the period: 9,628,185 Weighted average unit cost of PCR test during the period, estimated considering the evolution of the price of the PCR test in the NHS Conventions: €65.48 Investment in professional Ag-RDTs: Number of professional Ag-RDT conducted during the period, assuming they represented around 20,7% of the total tests performed in the period: 2,508,183 Weighted average unit cost of Ag-RDT, estimated considering the average market value price for the period under analysis: €20.00
Healthcare professionals	Time	–	The time required to perform the tests was considered as part of regular working hours.
Society	–	–	–
Total		680.6	

According to the calculated outcomes and inputs, the SROI ratio obtained was 1:8.2. This means that for €1 invested during the analyzed period, €8.2 of social value was generated. The findings indicate that IVD tests were not only critical in managing the public health crisis but also generated substantial returns across multiple domains – health, economic, and social. Notably, conservative assumptions were used (e.g., only one transmission prevented per positive case), suggesting that the actual value generated could be even greater.

To assess the robustness of the estimated SROI ratio, sensitivity analyses were performed on the most influential parameters of the model. First, varying the total investment value by ±20% (base value: € 680.6 million) resulted in SROI ratios ranging from €6.8 to €10.2 of social value generated per euro invested. This indicates that the SROI ratio is moderately sensitive to changes in total investment assumptions. Second, a ± 20% variation was applied to the outcome with the highest contribution to total impact, namely economic improvement (base value: €2,0471 million). Under this scenario, the SROI ranged between €7.6 and €8.8 per euro invested, showing a more limited sensitivity to variations in the valuation of this outcome.

In addition to the primary sensitivity analyses on investment and key outcomes, a complementary analysis was conducted to explore the impact of varying the monetary proxy assigned to QALYs. The monetary proxy for QALY varied between 10,000 and €100,000, reflecting the cost-effectiveness thresholds defined by Infarmed. Under this scenario, the SROI ratio ranged from €6.2 to €11.7, compared with the base-case SROI of €8.2 (proxy: €41,694 per QALY). This demonstrates that the conclusions regarding substantial social value creation remain robust, even when applying alternative QALY valuation thresholds.

Overall, the combined sensitivity analyses confirm that the COVID-19 intervention consistently generates social value, with the SROI ratio remaining well above 1:1 across plausible variations in both investment assumptions and health outcome variations.

### 3.2 Case study: glycemic control in diabetes management

This case study assessed the social impact of two key IVD tools—HbA1c and SMBG—in the management of type 1 and type 2 diabetes in Portugal. The analysis focused on a 1-year period (2019) and compared outcomes to a counterfactual scenario in which these monitoring tools were not used.

#### 3.2.1 Scope and stakeholders

The evaluation considered the widespread use of SMBG and HbA1c testing among patients treated in National Health System (NHS) primary care and hospital settings. The stakeholders included patients, as the primary beneficiaries of improved disease control and quality of life, families and caregivers, due to their role in supporting disease management, healthcare providers and system, which benefit from reduced complication rates and improved resource allocation, healthcare professionals, who gain from more effective treatment planning and greater job satisfaction, and society at large, through preserved productivity and reduced disability.

#### 3.2.2 Mapping outcomes of HbA1c and SMBG use

In 2019, healthcare providers performed roughly 1.25 million HbA1c tests on patients with both Type 1 and Type 2 diabetes. The majority of these tests—about 984,000—happened in primary care, while the remaining 261,000 took place in hospitals (17, 20). It is estimated that glycemic control through SMBG has been practiced by type I diabetics, by 40% of type II diabetics admitted to hospital and approximately 75% of diabetic patients followed up in primary health care (20), which corresponds to around 592,760 individuals with diabetes in Portugal.

The combination of these monitoring practices supported individualized treatment adjustments, improved glycemic control, and reduced acute chronic complications. This translated into multiple positive outcomes, including improved quality of life for patients, enhanced emotional, social and financial well-being for families, optimized healthcare costs and resource utilization, increased productivity through reduced absenteeism, and improved treatment planning and satisfaction among general practitioners. All outcomes derived from the expected chain of events triggered by glycemic monitoring are summarized by stakeholder group in Table 4.

**Table 4:** Diabetes impact map: quantity of change for outcomes by stakeholders.

Outcomes	Indicator	Number	Financial proxy	Adjustment	Impact calculation (€M)
Patients					
Improved quality of life (emotional, physical, and social wellbeing)	QALYs gained from SMBG (43)	7,009	WTP per capita [2x national GDP per capita (37)] *€41,682.00*	2% dwt 20% atb	228.9
Increased free time	Number of free days gained from reduced time spent on care by non-working diabetic patients	210,223	Average daily consumption per person *€ 12.00*	2% dwt 0% attb	2.5
Caregivers and/or family members					
Improved emotional well-being	No. of family members/ caregivers feeling emotionally better	725,210	Psychological Support (1 session per family member) *(€ 60.00)*	2% dwt 20% atb	34.1
Increased free time	No. of family days saved by not needing to attend medical appointments	21,022	Average daily consumption per person *€ 12.00*	2% dwt 0% atb	0.2
Increased household economic capacity due to reduced need for informal care	No. of diabetes patients avoiding severe complications and no longer needing caregiver	867	Annual household cost of a caregiver *€ 8,880.00*	2% dwt 20% atb	6.0
Healthcare system and providers					
Cost and resource optimization via reduced complication incidence	No. of severe complications avoided (20)	1,841	Weighted average Diagnosis Related Groups (DRGs) cost (20) *€ 3,457.17*	2% dwt 0% atb	6.2
	No. of patients no longer requiring rehabilitation (20)	1,841	Rehabilitation cost per patient (includes one consultation and four sessions per year) (25) *€ 54,43*	2% dwt 0% atb	0.1
	No. of diabetes patients avoiding dialysis	705	Comprehensive dialysis cost per patient *€ 23,589.28*	2% dwt 0% atb	16.3
Reduced costs and resources utilization via process optimization	No. of medical visits avoided due to glucose monitoring	725,210	NHS follow-up medical appointment cost (40) *€31.00*	2% dwt 0% atb	22.0
Healthcare professional					
Greater job satisfaction	No. of public primary care physicians (GPs)	5,575	Diabetes management training (1 day) *(€ 150.00)*	2% dwt 30% atb	0.6
Society					
Increased productivity	No. of work absence days avoided due to reduced medical visits among working-aged diabetes patients	125,059	Average daily cost per worker (41) *€ 90,87*	2% dwt 0% atb	11.1
Total					328.1

dwt, deadweight; atb, attribution.

#### 3.2.3 Evidencing outcomes and assigning value

Indicators for the diabetes case study were based on national health statistics, diabetes registry data, and international literature on glycemic control outcomes. For example, the increase in free time for individuals with diabetes was valued by estimating the total time saved (*n* = 210,233 days) and multiplying it by the average daily leisure consumption value per person (€12.00). The increase in household economic capacity among affected families was estimated based on the number of diabetic patients who avoided severe complications and consequently no longer required a caregiver (*n* = 867), monetized using the average annual caregiver cost (€8,880.00). The outcome related to healthcare professionals—specifically, improved job satisfaction—was quantified by the number of general practitioners working in primary care (*n* = 5,575) and valued using the cost of 1 day of annual diabetes management training (€150).

The indicators used to quantify each outcome, along their corresponding financial proxies, are presented in Table 4. Additional information on data sources and underlying assumptions can be found in Supplementary Table S3.

#### 3.2.4 Establishing impact

To determine the net benefit attributable to the use of HbA1c and SMBG, adjustments were made for deadweight (e.g., health improvements that might have occurred regardless of monitoring) and attribution (e.g., additional contributors such as dietary and therapeutic interventions). A full justification for these assumptions is provided in Supplementary Table S4.

#### 3.2.5 Calculating the SROI

The total investment in SMBG and HbA1c in 2019 was estimated at €54.8 million, encompassing costs related to test supplies (Table 5). After adjustments, the total social impact reached €328.1 million. The majority of the social value was generated through improved patient well-being (€228.9 million; 70%), followed by savings for the healthcare system (€ 44.6 million) and gains in family and caregiver well-being and support (Table 4).

**Table 5:** Diabetes: estimated inputs allocated to the use of HbA1c and SMBG.

Stakeholders	Resources	Value (€M)	Rationale
Patients	Time	–	The time spent learning how to use SMBG was deducted from the benefit associated with time saved from avoided medical appointments. Time spent on device use was not considered, as it represents a minimal unitary effort and is absorbed into daily routine activities. Time spent on HbA1c testing was not considered, as it is included in routine bloodwork for diabetic patients. The cost of glucose test strips borne by the patient is included under the healthcare system.
Caregivers and/or family members	Time	–	The time spent learning how to use SMBG was deducted from the benefit related to time saved from avoided appointments.
Healthcare System and Care Providers	Human resources, HbA1c testing, SMBG	54.8	HbA1c testing investment: Number of patients undergoing HbA1c testing twice annually at primary and secondary care (20) = approx. 1.2 million tests. Unit cost of HbA1c test (25): €7.30. SMBG investment: Cost of blood glucose test strips (17): €45.7 million.
Healthcare Professionals	Time	–	Time spent analyzing test results is considered part of the consultation time.
Society	–	–	–
Total		54.8	

The resulting SROI ratio was 1:6.0, indicating that for €1 invested in glycemic monitoring, €6 of social value was generated. The analysis confirms that systematic glycemic monitoring is a high-value intervention, with significant benefits for patients, their support networks, healthcare systems, and society at large. By facilitating better disease control and reducing the likelihood of severe complications such as renal failure, stroke, and cardiovascular events, these IVD tools play a crucial role in chronic disease management. Importantly, the estimated SROI is based on a conservative model and excludes unquantified benefits such as reductions in diabetic retinopathy due to the lack of reliable data to support them. Therefore, the true social return may be even greater than calculated.

To evaluate the robustness of the SROI estimate for the diabetes case study, sensitivity analyses were conducted on the most influential model parameters. A ± 20% variation in the total investment value (base value: €54.8 million) resulted in SROI ratios ranging from €5.0 to €7,5 of social value generated per euro invested, compared with the base case SROI of €6.0 per euro invested. This indicates a moderate sensitivity of the SROI ratio to assumptions related to investment costs. In addition, a ± 20% variation was applied to the outcome with the greatest contribution to total impact – improved patient well-being (base value: €228.9 million). Under this scenario, the SROI ratio ranged between €5,2 and €6,8 per euro invested, suggesting a relatively limited sensitivity to changes in the valuation of this outcome.

In addition, a complementary sensitivity analysis was performed by varying the monetary proxy assigned to QALY between €10,000 and €100,000, which resulted in SROI ratios between €2.8 and €11.8, compared with the base-case SROI of €6.0. These results indicate that, although the SROI estimate is sensitive to assumptions regarding the valuation of health outcomes, the intervention consistently generates positive social value across different QALY thresholds.

Overall, the combined sensitivity analyses confirm the robustness of the diabetes case study findings, with the SROI ratio remaining above 1:1 under all tested scenarios.

### 3.3 Case study: B-type natriuretic peptide testing for early diagnosis of heart failure

This case study evaluated the potential social return of prescribing the B-type natriuretic peptide test in primary care settings to support the early diagnosis of HF in Portugal. The analysis is based on a one-year scenario (2019), comparing the current diagnostic pathway—often characterized by delays and reliance on hospital-based imaging—with a more timely biomarker-based strategy aligned with European Society of Cardiology guidelines (23).

#### 3.3.1 Scope and stakeholders

The analysis considered the impact of conducting B-type natriuretic peptide tests on all patients aged ≥25 years presenting to primary care with symptoms suggestive of HF. The stakeholders included patients, as primary beneficiaries of faster diagnosis and improved disease control; families and caregivers, who experience changes in their emotional and economic burdens; the health system and providers, through cost optimization and better care planning; healthcare professionals, who experience improved clinical decision-making; and society, through reduced disability and productivity losses.

#### 3.3.2 Mapping outcomes of B-type natriuretic peptide test use

In 2019, an estimated 81,012 B-type natriuretic peptide tests would have been performed if prescribed in the Portuguese primary care setting for adults ≥ 25 years presenting with symptoms suggestive of HF. These tests would have resulted in the diagnosis of 42,190 patients with heart failure and the exclusion of 38,822 patients who did not have this syndrome. These estimates were based on the study of Fonseca et al. (21), a budget impact study about the use of NT-proBNP in primary settings during 2019.

The diagnostic clarification offered by the prescription of B-type natriuretic peptide tests in primary care reduces diagnostic delays and unnecessary referrals. Eleven outcomes were therefore included in this analysis. Four outcomes for patients, three for family relatives and/or caregivers, two for health system and providers and one for both healthcare professionals and society in general. The modeled outcomes included significant improvements in physical, emotional, and social well-being for patients, reduced caregiver burden and improved economic stability in affected families, health resource optimization and cost reduction, improved clinical workflow and satisfaction of general practitioners, and reduced absenteeism and increased productivity among working-age patients. All outcomes derived from the improved diagnostic pathway are detailed by stakeholder group in Table 6.

**Table 6:** HF impact map: quantity of change for outcomes by stakeholders.

Outcomes	Indicator	Number	Financial proxy	Adjustment	Impact calculation (€M)
Patients					
Improved emotional well-being	Number of patients with confirmed HF diagnosis feeling emotionally better (21)	27,719	Psychological support (1 session/month for 12 months) *€ 720.00*	38,6% dwt 10% atb	11.0
	No. of patients with HF ruled out who report feeling more at ease (21)	38,822	Psychological support (1 session/year) *€ 60.00*	32% dwt 40% atb	1.0
Improved physical well-being	No. of patients with confirmed HF diagnosis reporting improved physical condition	27,719	Physical activity program (Hydro 2/ week for 11 months) *€228.33*	38,6% dwt 10% atb	3.5
Improved social well-being	No. of patients with confirmed HF diagnosis reporting better quality of life and more availability for leisure activities	27,719	Average older person’s spending on leisure activities (38) *(€ 547.50)*	38,6% dwt 10% atb	8.4
Increased free time	No. of additional free days gained by non-working-age patients from avoided medical visits during diagnosis process	3,731	Average daily consumption per person *€ 12.00*	0% dwt 0% atb	0.04
Caregivers and/or family members					
Improved emotional well-being	No. of family members feeling emotionally more stable	27,719	Psychological support (1 session/month) *(€ 240.00)*	38,6% dwt 10% atb	3.7
Increased free time	No. of family members’ days gained from not attending medical appointments/ exams	933	Average daily consumption per person *€ 12.00*	0% dwt 0% atb	0.01
Increased economic capacity of affected families	No. of families no longer needing an active family member as caregiver	154	Annual difference between national minimum wage and informal caregiver allowance *€3,170.88*	38,6% dwt 10% atb	0.3
Healthcare system and providers					
Resource Optimization and Cost Reduction	No. of avoided medical appointments	5,620	NHS follow-up appointment cost (40) *(€ 31.00)*	0% dwt 0% atb	0.2
	No. of avoided echocardiograms	21,137	NHS echocardiogram cost (25) *€40.70*	0% dwt 0% atb	0.9
	No. of avoided hospitalizations	1,242	Hospitalization cost due to decompensated HF (24) *€1,430.07*	0% dwt 0% atb	1.8
Increase in treatment-related costs	No. of patients starting treatment earlier	25,905	Average cost of early-phase treatment/ patient (21) *(€ 33.52)*	0% dwt 0% atb	−0.9
Healthcare professional					
Greater Job Satisfaction	No. of general practitioners in public primary care reporting increased job satisfaction	5,575	HF patient management training (1 training/year – 4 h) *€ 60.00*	0% dwt 50% atb	0.2
Society					
Increased productivity	No. of work absence days avoided due to reduced medical visits, exams, and hospitalizations among working-age patients	3,519	Average daily cost per worker (41) (ages 25–64) *€ 66.63*	0% dwt 0% atb	0,2
Total					30.2

dwt, deadweight; atb, attribution.

#### 3.3.3 Evidencing outcomes and assigning value

This prospective analysis draws on national incidence estimates and modeled diagnostic outputs to assess the impact of introducing B-type natriuretic peptide prescription in primary care. Emotional well-being benefits were estimated for patients receiving an earlier and more accurate diagnosis of HF (*n* = 27,719), as well as for individuals for whom a diagnosis of HF was ruled out (*n* = 38,822). These benefits were valued using proxy measures based on individuals’ willingness to pay for equivalent emotional relief: one psychological consultation per month over a 12-month period (€720) for those diagnosed, and one consultation per year (€60) for those without the condition. Improvements in physical well-being were also estimated for patients with earlier and more accurate diagnoses (*n* = 27,719), using the annual cost of a monthly aqua aerobics course (€228.33) as a financial proxy. From the perspective of the healthcare system and providers, resource optimization and cost savings were assessed through reductions in medical appointments (*n* = 5,620), echocardiograms (*n* = 21,137), and hospital admissions (*n* = 1,242) attributable to earlier diagnosis. Unit costs were sourced from the NHS price list: €31.00 per consultation, €40.70 per echocardiogram (NHS-convention price), and €1,430.07 per hospitalization, based on DRG 194 for decompensated heart failure. On the other hand, the model accounted for increased treatment costs – an opposite outcome—resulting from the earlier initiation of therapy in 25,905 patients. The average treatment cost applied in the analysis was €33.52 per patient.

A comprehensive summary of outcomes, indicators, and proxies is provided in Table 6. Additional details regarding data sources and assumptions are available in Supplementary Table S5.

#### 3.3.4 Establishing impact

Adjustments were made for deadweight (e.g., users who start treatment for HF in the 1st month after their visit to the health center, regardless of the B-type natriuretic peptide test) and attribution (e.g., additional contributors such as patient support associations), in line with standard SROI practice. Conservative assumptions were applied, and details are provided in Supplementary Table S6.

#### 3.3.5 Calculating the SROI

The estimated investment for carrying out the B-type natriuretic peptide tests prescribed in primary care, in 2019, was €2.4 million, based on a €30 test unit cost (Table 7). The resulting total social value, after adjustments, was calculated at €30.2 million. The main divers of social value were improvements in patient quality of life and reductions in unnecessary resource utilization Table 6. The resulting SROI ratio was 1:12.6 – meaning that for €1 invested, €12.6 of social value could be generated.

**Table 7:** HF: estimated inputs allocated for the possible use of B-type natriuretic peptide test.

Stakeholders	Resources	Value (€M)	Rationale
Patients	Time	–	The time spent performing B-type natriuretic peptide tests was subtracted from the benefit related to time gained from avoided medical procedures.
Caregivers and/or family members	Time	–	The time spent accompanying patients for B-type natriuretic peptide tests was subtracted from the benefit related to time gained from avoided medical procedures.
Healthcare System and Care Providers	Human resources, B-type natriuretic peptide test	2.4	Investment in B-type natriuretic peptide tests: Number of patients presenting primary care with symptoms suggestive of HF and eligible for B-type natriuretic peptide testing (21): 81,012 Cost per B-type natriuretic peptide test (24): €29.60
Healthcare Professionals	Time	–	The time required to perform B-type natriuretic peptide tests was considered part of the professionals’ normal working hours.
Society	–	–	–
Total		2.4	

The findings suggest that implementing B-type natriuretic peptide prescription in primary care for patients with suspected HF offers substantial value, enabling earlier and more accurate diagnoses, avoiding unnecessary procedures, and improving treatment outcomes. The projected return on investment is particularly significant given the relatively low cost of implementation. Notably, the SROI estimate is conservative and does not account for all potential benefits—such as gains in life expectancy or avoided long-term complications—implying that the real impact could be even greater.

To assess the robustness of the SROI estimate for the heart failure case study, sensitivity analyses were conducted on key model parameters. A ± 20% variation in the total investment value (base value: €2.4 million) resulted in SROI ratios ranging from €10.5 to €15.7 of social value generated per euro invested, compared with the base-case SROI of €12.6 per euro invested. This indicates a moderate sensitivity of the SROI ratio to changes in investment assumptions. Furthermore, a ± 20% variation was applied to the outcome with the largest contribution to total impact, namely improved emotional well-being of patients and family members (base value: €15.7 million). Under this scenario, the SROI ratio ranged between €11.3 and €13.9 per euro invested, suggesting a relatively limited sensitivity to variations in the valuation of this outcome. In general, the sensitivity analysis demonstrates the SROI results for the heart failure case study remain robust across plausible variations in key parameters, with the SROI ratio consistently remaining above 1:1.

### 3.4 Case study: molecular testing in advanced non-small cell lung cancer

This case study evaluated the SROI of using NGS to guide treatment in patients newly diagnosed with stage IV lung adenocarcinoma, in Portugal. The analysis covered 1 year (2019) and compared two scenarios: one in which molecular testing informs target therapy selection, and another where only standard chemotherapy is administered due to the absence of genetic testing.

#### 3.4.1 Scope and stakeholders

The analysis included all patients diagnosed with stage IV adenocarcinoma and eligible for NGS testing (19). The stakeholders considered were patients, the ones that benefit from personalized therapies with greater efficacy and fewer side effects, families and caregivers, who experience lower care burdens and improved emotional stability, healthcare systems and providers, impacted by both reduced use of toxic chemotherapy and increased medication costs, healthcare professionals, whose treatment decisions are improved by biomarker data, and society, due to gains in productivity and prolonged, higher-quality life among patients.

#### 3.4.2 Mapping outcomes of NGS molecular testing use

Given the number of new cases of lung cancer in Portugal in 2019 (5,208 (19)), and assuming that 85% are Non-Small Cell Lung Carcinoma (NSCLC), and of these, 57% (29) are adenocarcinoma and around 57.1% (30) are stage IV, around 1,441 new cases of stage IV adenocarcinoma are estimated. According to national references, around 89% of these cases are submitted to an evaluation of the mutational status (29), which means that it is estimated that, in 2019, 1,282 patients newly diagnosed with stage IV adenocarcinoma of the lung underwent NGS testing to identify actionable mutations. Based on international evidence, around 54% (692 patients) were found to have actionable mutations and were eligible for target therapies (30).

These outputs led to the inclusion of fourteen key outcomes, six for lung cancer patients, three for patients’ relatives and/or caregivers, three for health system and providers and one for both health professionals and society. The main outcomes are related to increased survival (an average of 1.45 years gain per treated patient), better physical, emotional, and social well-being, fewer complications and hospital visits related to chemotherapy, increased treatment cost, improved work capacity and reduced caregiver burden, and higher professional satisfaction due to improved treatment precision. The full set of outcomes by stakeholders is presented in Table 8.

**Table 8:** Lung cancer impact map: quantity of change for outcomes by stakeholders.

Outcomes	Indicator	Number	Financial proxy	Adjustment	Impact calculation (€M)
Patients					
Increased survival for patients with stage IV lung adenocarcinoma	Number of patients with increased survival	692	Value of 1.45 (30) additional life years for a lung cancer patient *(€34,462.44)*	0% dwt 0% atb	23.9
Improved emotional well-being	No. of patients who feel more positive due to eligibility for targeted therapy	692	Psychological support (2 sessions/month) *€1,440.00*	5% dwt 10% atb	0.9
	No. of patients feeling emotionally better due to avoiding aggressive treatments	692			0.9
Improved physical well-being	No. of patients maintaining physical capacity	692	Gym membership (annual) *€ 440.87*	5% dwt 0% atb	0.3
	No. of patients no longer requiring concomitant treatments due to lower toxicity	692	Cost of antiemetics and iron supplements *€ 193.87*	5% dwt 0% atb	0.1
Improved social well-being	No. of lung cancer patients feeling able to engage in social and leisure activities	692	Annual average leisure spending per adult (38) *€ 1,260.50*	5% dwt 0% atb	0.8
Reduced complications from IV therapy	No. of instances of IV-related complications (e.g., bruising)	415	Cost of managing complications (thrombocid, oral diclofenac) *(€ 14.79)*	10% dwt 0% atb	0.01
Increased free time	No. of non-working patients with more free time due to less time in treatment	432	Average daily spending per person during free time *€ 12.00*	5% dwt 0% atb	0.00
Caregivers and/or family members					
Improved emotional well-being	No. of family members feeling emotionally more stable	692	Psychological support (2 sessions/month) *(€ 1,440.00)*	5% dwt 10% atb	0.9
Improved social well-being	No. of family members who maintain leisure activities due to patient stability	692	Annual average leisure spending per adult (38) *€ 1,260.50*	5% dwt 0% atb	0.8
Reduced burden of formal/Informal care	No. of family members no longer needing to provide or hire care	692	Annual cost of caregiving to the household *(€ 8,880.00)*	5% dwt 10% atb	5.3
Healthcare system and providers					
Reduced chemotherapy needs	No. of avoided chemotherapy sessions (6 per patient)	4,155	Cost per chemotherapy session (24) *(€ 496.30)*	0% dwt 0% atb	2.1
Improved patient safety due to fewer adverse events	No. of patients potentially avoiding chemo-related adverse events	21	Annual average cost of adverse events (44) *€ 92,907.00*	5% dwt 0% atb	1.8
Increased treatment costs	No. of lung cancer patients receiving targeted therapy	692	Annual cost of targeted therapy/ patient *(€ 48,285.72)*	0% dwt 0% atb	−33.4
Healthcare professional					
Greater job satisfaction	No. of oncologists and pulmonologists reporting greater satisfaction	348	Oncology conference participation *€ 753.56*	21% dwt 10% atb	0.2
Society					
Increased productivity during treatment	No. of working-age patients maintaining employment during treatment	260	Average annual cost per worker (41) *€ 20,899.84*	5% dwt 0% atb	5.2
Total					9.6

dwt, deadweight; atb, attribution.

#### 3.4.3 Evidencing outcomes and assigning value

The indicators used in this analysis were derived from national cancer registry data (RON), relevant epidemiological studies, and international evidence on the effectiveness of targeted therapies in stage IV lung adenocarcinoma. For instance, improvements in patients’ social well-being were estimated by assuming that the 692 individuals likely to benefit from targeted therapies would be able to continue participating in social and leisure activities. This outcome was monetized using 50% of the average annual expenditure on leisure activities by an adult (€1,260.50). Still from the patient’s perspective, a reduction in complications associated with the administration of intravenous therapy was also considered. This outcome was estimated based on the number of instances in which complications such as bruising may occur during IV medication administration (*n* = 415) and was monetized using the market cost of typical treatments employed to mitigate such effects (€14.79). From the perspective of the healthcare system and service providers, a reduction in chemotherapy demand was also considered. This was calculated based on six avoided chemotherapy cycles per patient (totaling 4,155 sessions), using the average unit cost per session as defined by the DRG system (€496.30).

A detailed overview of the indicators and financial proxies applied to each outcome—including gains in survival, and productivity—is presented in Table 8. Additional information regarding data sources and methodological assumptions can be found in Supplementary Table S7.

#### 3.4.4 Establishing impact

Adjustments were applied for deadweight (e.g., The occurrence of complications during the administration of IV therapy, for instance, also depends on the condition and physical characteristics of the patient and the administration environment) and attribution (e.g., additional contributors such as cancer patient associations), while also accounting for increased treatment costs associated with target therapies (negative outcome). Detailed justifications are presented in Supplementary Table S8.

#### 3.4.5 Calculating the SROI

The total investment in NGS testing for eligible patients in 2019 was €0.64 million, based on a €500 unit cost (Table 9). After excluding external influences (attribution and deadweight), the total estimated social value generated was €9.57 million. The largest share of social value came from extended survival and improved quality of life for patients (€26.8 million), although offset by an increase in system costs due to higher drug prices.

**Table 9:** Lung cancer: estimated inputs allocated to molecular testing.

Stakeholders	Resources	Value (€M)	Rationale
Patients	Time	–	It was considered that the time required for the collection of biological material for genetic alteration testing (NGS) is included within the consultation time.
Caregivers and/or family members	Time	–	It was considered that the time spent accompanying patients for the collection of biological material for genetic alteration testing (NGS) is included within the consultation time.
Healthcare System and Care Providers	Human resources, NGS panel	0.64	Investment value in the NGS Panel: Number of stage IV adenocarcinoma cases diagnosed and tested using the NGS panel: 1,282 Market price for performing the NGS panel per patient: €500.00
Healthcare Professionals	Time	–	It was considered that the time required to perform biopsies for sample collection is included within regular working hours.
Society	–	–	–
Total		0.64	

The final SROI ratio was 1:14.9, meaning that for €1 invested in NGS testing, €14.9 of social value was generated. The results demonstrate that investing in molecular IVD tests for stage IV adenocarcinoma enables more effective and less toxic treatment pathways, significantly improving clinical and quality-of-life outcomes. This also contributes to the optimization of healthcare resource use and increased societal productivity, particularly in working-age patients. Despite the increase in drug-related costs, the benefits in reduced complications, improved outcomes, and enhanced patient autonomy outweighs the initial investment. Moreover, since this SROI estimate only includes quantifiable benefits, the real return on investment is likely even higher.

To evaluate the robustness of the SROI estimate for the lung cancer case study, sensitivity analyses were conducted on key model parameters. A ± 20% variation in the total investment value (base value €0.64 million) resulted in SROI ratios ranging from €12.4 to €18.7 of social value generated per euro invested, compared with the base-case SROI of €14.9 per euro invested. This indicates a moderate sensitivity of the SROI ratio to changes in investment assumptions. In addition, a ± 20% variation was applied to the outcome with the greatest contribution to total impact, namely increased survival for patients with stage IV lung adenocarcinoma (base value: €23.8 million). Under this scenario, the SROI ration ranged between €7.5 and €22.4 per euro invested, indicating a higher sensitivity of the SROI estimate to variations in the valuation of this outcome.

Overall, despite the greater sensitivity observed for the main outcome, the sensitivity analysis confirms that the lung cancer case study generates substantial social value across all tested scenarios, with the SROI ratio remaining consistently above 1:1.

## 4 Discussion

### 4.1 Interpretation of main findings

The findings from the four case studies presented in this research strongly reinforce the significant economic and social value generated by IVD tests across a diverse range of clinical conditions. Whether applied in infectious diseases, chronic conditions, heart failure, or advanced cancer, these diagnostic tools consistently demonstrated high returns on investment - ranging from €6.0 to nearly €15 in social value for every €1 invested, illustrating that even modest investments in IVD tests can yield substantial and multifaceted benefits.

Each case study demonstrates how a specific IVD test contributes to improved patient care and enhanced health system performance. In the context of the COVID-19 pandemic, the use of molecular and antigen testing (PCR and Ag-RDTs) enabled early detection and effective isolation of cases, helping to reduce transmission, ease the burden on hospitals and lower mortality rates. The estimated return of €8.2 per euro invested illustrates the critical role of IVD tests in public health crises.

In chronic disease management, routine HbA1c testing and self-monitoring helped both patients and healthcare teams in preventing complications and hospitalizations, yielding a return of €6.0 for every euro invested. While this return is lower than in other case studies, it reflects the long-term nature of chronic conditions and the more gradual, diffused benefits over time.

In the field of HF diagnosis, the hypothetical use of B-type natriuretic peptide testing after prescription in primary health care setting—where such testing has historically been underutilized – showed an estimated return of €12.6 for each euro invested, suggesting a significant opportunity to optimize care pathways through earlier and more accurate diagnosis.

Finally, the application of NGS panel in patients with stage IV lung adenocarcinoma, in line with the current guidelines, enabled the selection of more personalized therapies, significantly improving both survival outcomes and quality of life while delivering a high return (€14.9). These results highlight the transformative impact of precision diagnostics in oncology.

Together, these findings reveal a common theme: despite representing a relatively small share of overall healthcare spending, IVD tests delivery value that extends far beyond their immediate diagnostic role. They contribute to better clinical decisions, reduce unnecessary interventions, help prevent disease progression, and ultimately enhance quality of life, productivity and survival. Moreover, the benefits of diagnostic information extend across a broad range of stakeholders, including patients, families and caregivers, healthcare professionals, providers and healthcare system, and society as a whole. Importantly, the use of the SROI methodology enabled the quantification of indirect and intangible outcomes—such as emotional well-being, personalized treatment, and caregiver burden relief—that are often omitted in conventional health economic evaluations. Furthermore, these findings were shown to be robust to plausible variations in key assumptions, as confirmed through sensitivity analyses conducted across all case studies.

### 4.2 Strengths and limitations

To the best of our knowledge, this is the first SROI study applied to IVD tests in four different clinical contexts. This study has several strengths. First, it applied a standardized, transparent, and stakeholder-centered methodology (SROI), enabling a multidimensional assessment of value. Second, by examining four distinct clinical areas, the study enhances the robustness and transferability of the findings. Third, the analyses used real-world data and nationally relevant assumptions, incorporating conservative estimates and adjusting for deadweight and attribution. Finally, the inclusion of outcomes such as caregiver burden, quality of life, and workforce productivity reflects a strong commitment to patient-centered and socially oriented evaluation.

An additional strength of this study lies in the explicit assessment of uncertainty through sensitivity analyses. By varying key model parameters by ±20%, namely total investment values and the outcomes with the greatest contribution to total impact, the analysis demonstrated that the SROI ratios remained consistently above 1:1 across all four case studies. While variations in investment assumptions led to moderate changes in SROI estimates, greater sensitivity was observed when high-impact health outcomes, such as survival gains in advanced lung cancer or quality-of-life improvements were varied. This reflects the substantial weight of these outcomes in driving social value estimates and underscores the importance of transparent assumptions when monetizing complex health benefits.

An additional sensitivity assessment was performed to explore the impact of varying the monetary proxy assigned to QALYs in COVID-19 and diabetes case studies. Across plausible thresholds [from €10,000 to €100,000 per QALY, based on Infarmed guidance (28)] SROI estimates remained robust, with variations in the ratio demonstrating the resilience of the findings to different assumptions regarding the value of health gains.

Overall, the sensitivity analyses reinforce the robustness of the findings while appropriately acknowledging the inherent uncertainty associated with modeling-based evaluations.

The application of the SROI methodology in this study aligns with a growing body of evidence supporting its relevance in healthcare evaluation. Previous SROI analyses have demonstrated its utility in assessing interventions across a wide range of clinical contexts. For instance, a Spanish study on the management of heart failure in the national health system applied SROI to a set of 28 proposals – including case management, cardiac rehabilitation, and psychological support – and estimated a return of €3.52 for each euro invested, highlighting system-wide and patient-level benefits (31). Similarly, a recent Australian study on device-aided therapies (DATs) for advanced Parkinson’s disease showed that every AUD $1 invested generated AUD $1.79 in social value, most of which was related to emotional well-being and improved quality of life for patients and their families (32).

Other studies from the UK further corroborate this evidence. The MY LIFE program for type II diabetes management, in Wales, reported a return of £4.23–£5.07 per £1 invested (33), and the Westbank Living Well, Taking Control (LWTC) community-based diabetes prevention and management education program yielded an SROI of £5.80 during a three-year period (34). Together, these studies illustrate how SROI can provide a more comprehensive and socially relevant assessment of healthcare value, particularly when considering outcomes that are not captured in traditional cost-effectiveness analyses.

However, this study also has several limitations that should be acknowledged. The analysis of the quantified data relied on modeled outcomes and secondary data rather than primary patient-level information, which may affect the accuracy of the estimates. From a prudent perspective, only monetizable outcomes were included in the SROI calculations, likely leading to an underestimating of the total value generated.

The one-year time horizon may not fully capture the cumulative long-term benefits, particularly for chronic or progressive diseases such as diabetes, where health gains accumulate over extended periods. Nevertheless, this timeframe was intentionally selected to reflect the assessment of IVD tests that are already established and used in clinical practice, rather than newly introduced technologies. As such, the analysis focuses on the incremental and observable impacts within a defined period, providing a conservative estimate of value while avoiding the attribution of speculative long-term effects.

Moreover, while the analysis was tailored to the Portuguese context, differences in health systems, pricing, and population dynamics may limit the generalizability of the results. It should also be noted that, as IVD tests are embedded within broader clinical pathways, it remains challenging to fully isolate their individual contribution from the impact of subsequent interventions.

Finally, it is important to acknowledge that, although this study received financial support from an industry association, the funder had no role in the study design, data collection, analysis, interpretation of results, or manuscript preparation. Methodological transparency, conservative assumptions and sensitive analyses were applied to safeguard analytical independence.

### 4.3 Implications for practice and policy

As the aim of this study is not to inform funding or reimbursement decisions, but rather to evaluate the social value generated by IVD tests in Portugal, SROI constitutes an appropriate methodological approach. By allowing a structured estimation of the value created for multiple stakeholders, this approach provides insights into the transformative potential of IVD testing for public health, society, and the economy.

The findings offer actionable insights for decision-makers, health technology assessors, and policy developers. Firstly, they underscore the need to incorporate broader value frameworks into health technology assessment processes, especially for IVD tests, and highlight the potential of SROI as a practical tool for capturing system-wide value in a way that is both accessible and relevant to stakeholders across the health and economic sectors.

This perspective is particularly relevant considering the new European regulation on HTA and the forthcoming implementation of Joint Clinical Assessment (JCAs), including high-risk technologies such as IVDs. These procedures will require increasingly robust, harmonized clinical evidence at the European level, potentially prioritizing traditional clinical effectiveness, performance, and cost-effectiveness metrics in decision-making processes. While such requirements are essential to ensure scientific rigor and comparability, they may not fully capture the broader social, organizational, and system-level benefits generated by diagnostic technologies.

In this context, SROI should not be viewed as a substitute for cost-effectiveness analyses or clinical performance studies, but rather as a complementary approach that provides additional insights into value domains that are often underrepresented or difficult to quantify within conventional HTA frameworks. By integrating clinical, economic, and social outcomes, SROI can support a more holistic understanding of the contribution of IVD tests to healthcare systems, informing strategic planning, prioritization, and investment decisions alongside established HTA methodologies.

As healthcare systems evolve toward value-based care, IVD tests should no longer be viewed as ancillary services, but rather as foundational enablers of effective, equitable, and sustainable healthcare. Investing in IVD tests is not only clinically sound, but also economically and socially strategic. This analysis also highlights a broader imperative: the need to enhance the integration of diagnostic testing into strategic planning for health innovation and sustainability. Beyond their role in improving patient care, IVD tests also serve as foundational tools in research and development. Their role in supporting the development of target therapies and the understanding of emerging diseases underlines their importance in advancing medical progress.

In conclusion, this study demonstrates that IVD testing represents a high-impact, relatively low-cost intervention with substantial potential to improve individual and population health outcomes, optimize healthcare resource utilization, and generate substantial social and economic value. While recognizing the central role of clinical and economic evidence in future European HTA and JCA processes, this analysis highlights the importance of developing complementary business cases that articulate the full return on investment of IVD tests, from a holistic perspective. Such approaches are essential to ensure that the broader value of diagnostics is adequately recognized within increasingly standardized and evidence-driven regulatory environments.

## Data Availability

The original contributions presented in the study are included in the article/Supplementary material, further inquiries can be directed to the corresponding author.

## Author’s note

This article was developed with reference to the white paper “*Valor dos Testes Diagnóstico In Vitro (DIV) na Saúde, Sociedade e Economia*” published by Apifarma (35), which provided a foundational framework for the present analysis.
