# Supplementary material for: Impact of in vitro diagnostic tests in health, society and economy in Portugal
Source: Front Public Health. 2026 Feb 20;14:1715491. doi: 10.3389/fpubh.2026.1715491 (PMC12963253; doi:10.3389/fpubh.2026.1715491)
Supplement: Supplementary file 1 [file Table_1.docx]

Supplementary Material

Impact of In Vitro Diagnostic (IVD) Tests in Health, Society and Economy in Portugal

Paula Rodrigues^1^, Sílvia Mota^1^, Carolina Amaral^1^, Bruno Garganta^2^, Carlos Catalão^3^, Liliana de Almeida^3^, Inês Teixeira^4*^, Paula Costa^4,5^, Paulo Dias^4^

^1^Antares Consulting, Lisbon, Portugal

^2^ Werfen, Lisbon, Portugal

^3^ Roche Sistemas de Diagnósticos, Lisbon, Portugal

^4^ Portuguese Pharmaceutical Industry Association – APIFARMA, Lisbon, Portugal

^5^ Comprehensive Health Research Centre, Universidade de Évora, 7000-811 Évora, Portugal

*** Correspondence:**Inês Teixeira
ines.teixeira@apifarma.pt

# Supplementary Data

## Details about COVID-19 Case Study

Table S1: Quantities and valuation of outcomes in the COVID-19 Case Study

| Outcomes | Indicators | Quantity (a) | Outcome valuation (b) | Value | Rationale |
| --- | --- | --- | --- | --- | --- |
| PATIENTS AND FAMILIES | | | | | |
| Improved quality of life due to serious sequelae and avoided deaths | QALYs gained through avoided morbidity and mortality (1) | 68,599 | WTP per capita (2x national GDP per capita (30)) | €41,694.0 | **(a)** Quality of life improvement is based on an average gain of 2.78 QALYs from avoided morbidity (applicable to severe sequelae) and 2.92 QALYs from avoided mortality, according to the literature (1). Assuming that IVD tests helped avoid at least one infection per infected individual during the period, the estimated avoided infections are 837,416. QALYs are assigned to patients and families who were not infected and thus did not develop severe sequelae or die. Main assumptions include:  - 8,374 severe sequelae avoided (1% of COVID-19 cases) (1).  - 15,520 deaths avoided (based on a 1.9% average mortality rate in Portugal).  **(b)** QALYs are valued using society’s willingness to pay (WTP), set at 2x Portugal’s 2021 GDP per capita (€20,847 (2)). |
| Improved physical well-being | Number of mild symptomatic COVID-19 cases avoided | 394,814 | Physical activity | €36.74 | **(a)** Estimated from the avoided cases (n=837,416), assuming 50% were asymptomatic (3), subtracting the avoided deaths (n=15,520) and severe sequelae (n=8,374).  **(b)** Monthly gym membership (per case) obtained according to market prices. |
| Improved social well-being | Number of asymptomatic and mild symptomatic COVID-19 cases avoided | 813,522 | Average monthly adult spending on leisure activities | €210.08 | **(a)** Total avoided infections (n=837,416) minus avoided deaths (n=15.520) and severe sequelae (n=8.374), considering a similar behavior of avoided COVID-19 cases with those registered in the period.  **(b)** Monthly leisure spending per adult, considering an annual expenditure of €2,521 (4). |
|  | Number of negative COVID-19 tests providing social well-being due to infection exclusion | 10,461,536 | Average biweekly adult spending on leisure activities | €105.04 | **(a)** Negative tests calculated as total tests (n=12,136,368 (5)) minus estimated positive test cases, assuming 2 tests per positive case (n= 837,416 × 2), which represents 1 confirmation test for each positive case.  **(b)** Biweekly leisure spending per adult, considering an annual expenditure of €2,521 (4) and 24 weeks. |
| Increased sense of security | Number of COVID-19 cases identified who adopted measures to avoid further infections | 837,416 | Psychological support | €60.00 | **(a)** Number of COVID-19 cases detected via PCR and professional-use rapid antigen tests (5).  **(b)** One psychology session per positive case, considering market prices (€60). |
|  | Number of negative COVID-19 tests that provided tested individuals a sense of safety due to infection exclusion | 10,461,536 | Psychological support | €60.00 | **(a)** Negative tests calculated as total tests (n=12,136,368 (5)) minus estimated positive test cases, assuming 2 tests per positive case (n= 837,416 × 2), which represents 1 confirmation test for each positive case.  **(b)** One psychology session per negative case, considering market prices (€60). |
| HEALTH SYSTEM / PROVIDERS | | | | | |
| Reduced hospitalization burden | Number of hospitalization days (including ICU) avoided | 600,164 | Average daily cost of COVID-19 hospitalization | €643.86 | **(a)** Avoided hospitalization days estimated using observed data, assuming avoided cases behave similarly to observed cases (6).  **(b)** Average COVID-19 hospitalization cost (€8,177) divided by average length of stay (12.7 days) (7). |
| Reduced burden of long COVID care | Number of avoided medical appointments due to reduction in Long COVID cases | 167,483 | NHS follow-up medical appointment cost | €31.00 | **(a)** Two visits avoided per Long COVID case avoided, assuming 10% of COVID-19 cases develop Long COVID.  **(b)** The cost of a follow-up medical appointment according to the NHS price list (8). |
| HEALTHCARE PROFESSIONALS | | | | | |
| Improved workplace safety and well-being | Number of tests (positive or negative) performed on healthcare workers | 1,495,224 | Psychological support | €60.00 | **(a)** Average of 2 tests/month for 62,301 doctors and nurses in Portuguese Public Hospitals (2021) (9).  **(b)** One psychology session per test, considering market prices (€60). |
| SOCIETY | | | | | |
| Increased productivity | Number of work absence days avoided due to reduced hospitalizations in the working-age population | 347,180 | Average daily cost per worker | €59.20 | **(a)** Hospitalization days avoided, assuming a percentage of 58% of hospitalizations in the 18-64 age group (7).  **(b)** Daily labor cost based on 14 months of average salary (€1,247.21/month (10)) plus employer contributions (23.75%), divided by 365. |
|  | Number of work absence days avoided due to reduced isolation in the working-age population | 2,322,317 | Average daily labor cost | €59.20 | **(a)** 10 isolation days avoided per infection prevented, adjusted for working-age population (60%) and remote work capacity (50%), subtracting 209,110 days spent on testing.  **(b)** See rationale above. |
| Economic improvement | Estimated number of lockdown months avoided | 3 | Difference between average GDP during lockdown and post-lockdown periods | €947,741,666.67 | **(a)** Months of lockdown avoided due to testing, leading to an early end of state of emergency in May 2020.  **(b)** GDP difference: €17.24B (post-lockdown) vs. €16.29B (during lockdown) (11). |

Table S2: Adjustments and impact determination in the COVID-19 Case Study

| Outcomes | Indicators | Deadweight (a) | Attribution (b) | Impact | Rationale |
| --- | --- | --- | --- | --- | --- |
| PATIENTS AND FAMILIES | | | | | |
| Improved quality of life due to serious sequelae and avoided deaths | QALYs gained through avoided morbidity and mortality (1) | 20% | 25% | €1,716,089,136.46 | **(a)** Percentage (%) of benefit estimated to be achieved using self-tests.  **(b)** % attributed to personal protective equipment and other preventive measures. |
| Improved physical well-being | Number of mild symptomatic COVID-19 cases avoided | 20% | 25% | €8,702,980.18 | **(a)** % of benefit estimated to be achieved using self-tests.  **(b)** % attributed to personal protective equipment and other preventive measures. |
| Improved social well-being | Number of asymptomatic and mild symptomatic COVID-19 cases avoided | 20% | 25% | €102,544,427.93 | **(a)** % of benefit estimated to be achieved using self-tests.  **(b)** % attributed to personal protective equipment and other preventive measures. |
|  | Number of negative COVID-19 tests providing social well-being due to infection exclusion | 20% | 10% | €791,205,967.68 | **(a)** % of benefit estimated to be achieved using self-tests.  **(b)** % attributed to personal protective equipment and other preventive measures. |
| Increased sense of security | Number of COVID-19 cases identified who adopted measures to avoid further infections | 20% | 10% | €36,176,371.20 | **(a)** % of benefit estimated to be achieved using self-tests.  **(b)** % attributed to personal protective equipment and other preventive measures. |
|  | Number of negative COVID-19 tests that provided tested individuals a sense of safety due to infection exclusion | 20% | 10% | €451,938,355.20 | **(a)** % of benefit estimated to be achieved using self-tests.  **(b)** % attributed to personal protective equipment and other preventive measures. |
| HEALTH SYSTEM / PROVIDERS | | | | | |
| Reduced hospitalization burden | Number of hospitalization days (including ICU) avoided | 20% | 25% | €231,852,332.03 | **(a)** % of benefit estimated to be achieved using self-tests.  **(b)** % attributed to personal protective equipment and other preventive measures. |
| Reduced burden of long COVID care | Number of avoided medical appointments due to reduction in Long COVID cases | 20% | 25% | €3,115,187.52 | **(a)** % of benefit estimated to be achieved using self-tests.  **(b)** % attributed to personal protective equipment and other preventive measures. |
| HEALTHCARE PROFESSIONALS | | | | | |
| Improved workplace safety and well-being | Number of tests (positive or negative) performed on healthcare workers | 20% | 10% | €64,593,676.80 | **(a)** % of benefit estimated to be achieved using self-tests.  **(b)** % attributed to personal protective equipment and other preventive measures. |
| SOCIETY | | | | | |
| Increased productivity | Number of work absence days avoided due to reduced hospitalizations in the working-age population | 20% | 25% | €12,331,786.64 | **(a)** % of benefit estimated to be achieved using self-tests.  **(b)** % attributed to personal protective equipment and other preventive measures. |
|  | Number of work absence days avoided due to reduced isolation in the working-age population | 20% | 25% | €82,488,373.12 | **(a)** % of benefit estimated to be achieved using self-tests.  **(b)** % attributed to personal protective equipment and other preventive measures. |
| Economic improvement | Estimated number of lockdown months avoided | 20% | 10% | €2,047,122,000.00 | **(a)** % of benefit estimated to be achieved using self-tests.  **(b)** % attributed to personal protective equipment and other preventive measures. |
| Total Impact |  |  |  |  | **€5,548,160,594.77** |

## Details about Diabetes Case Study

Table S3: Quantities and valuation of outcomes in the Diabetes Case Study

| Outcomes | Indicators | Quantity (a) | Outcome valuation (b) | Value | Rationale |
| --- | --- | --- | --- | --- | --- |
| PATIENTS | | | | | |
| Improved Quality of Life (emotional, physical, and social well-being) | QALYs gained from SMBG | 7,009 | WTP per capita | €41,682.00 | **(a)** Based on an average QALY gain of 0.04 for patients managing their chronic condition via SMBG, according to the literature (12). The estimated 175,230 type I and II diabetes patients benefiting are based on (13,14):  - 65% of type II patients in primary care use SMBG, and 10% act on results.  - 40% of type II patients in hospitals use SMBG, with 25% acting on results.  - 100% of type I patients in hospitals use SMBG.  **(b)** QALY is valued at 2x Portugal's 2019 GDP per capita (€20,841 (2)). |
| Increased free time | Number of free days gained from reduced time spent in care for non-active patients | 210,223 | Average daily consumption per person | €12.00 | **(a)** 0.5 days per medical appointment avoided (n=725,210), considering 62.7% of patients are not in the workforce (15), subtracting training time for SMBG.  **(b)** Average cost of a meal, considering market prices. |
| FAMILY MEMBERS AND/OR CAREGIVERS | | | | | |
| Improved emotional well-being | Number of family members/caregivers feeling emotionally better | 725,210 | Psychological support | €60.00 | **(a)** One caregiver per type II patient in primary care (n=656,265) and one per type I patient in hospitals (n=68,945) (13,14).  **(b)** One annual psychology session**,** considering market prices (€60). |
| Increased free time | Number of family members/caregiver days saved by not needing to attend medical appointments | 21,022 | Average daily consumption per person | €12.00 | **(a)** 10% of the free days gained by non-active patients (n=210,223), assuming 10% requires a companion.  **(b)** Average cost of a meal, considering market prices. |
| Increased household economic capacity due to reduced need for informal care | Number of diabetes patients avoiding severe complications and no longer needing caregiver | 867 | Annual household cost of a caregiver | €8,880.00 | **(a)** 50% of patients who suffer a stroke (n=1,258), 40% of those who suffer a myocardial infarction (MI) (n=556) and 100% of those who suffer an amputation (n=16) (14) are incapacitated for at least a year, requiring care.  **(b)** Average annual household expenditure on informal care, considering market values. |
| HEALTH SYSTEM / PROVIDERS | | | | | |
| Cost and resource optimization via reduced complication incidence | Number of severe complications avoided | 1,841 | Weighted average Diagnosis Related Group (DRG) cost | €3,457.17 | **(a)** Reduction in the incidence of some serious complications of diabetes, namely: transplants (n=12), strokes (n=1,258), MIs (n=556), and amputations (n=16), attributed to glycemic control (14)**.**  **(b)** Weighted average DRG cost based on complication types (14). |
|  | Number of patients no longer requiring rehabilitation | 1,841 | Rehabilitation cost per patient | €54.43 | **(a)** All complication cases assumed to require physical rehab during recovery.  **(b)** Estimated annual cost per patient includes one physiatry visit and four rehab sessions (16). |
|  | Number of diabetes patients avoiding dialysis | 705 | Comprehensive dialysis cost per patient | €23,589.28 | **(a)** From 881 new dialysis cases in 2019 with diabetes as primary condition, 80% attributed to DIV tests’ preventive impact (14).  **(b)** Weekly dialysis cost (€453.64) annualized. |
| Reduced costs and resources utilization via process optimization | Number of medical visits avoided due to glucose monitoring | 725,210 | NHS follow-up medical appointment cost | €31.00 | **(a)** At least one annual visit avoided per type II patient in primary care and type II patient in hospitals (13).  **(b)** The cost of a follow-up medical appointment according to the NHS price list (8). |
| HEALTHCARE PROFESSIONALS | | | | | |
| Greater job satisfaction | Number of public primary care physicians (GPs) | 5,575 | Diabetes management training | €150.00 | **(a)** GPs working in primary care.  **(b)** One annual training day in Diabetes management, according to market values. |
| SOCIETY | | | | | |
| Increased productivity | Number of work absence days avoided due to reduced medical visits among working-aged diabetes patients | 125,059 | Average daily cost per worker | €90.87 | **(a)** 0.5 days per avoided medical appointments (n=725,210), for 37.3% of working-age patients (15), minus time spent learning SMBG.  **(b)** Based on 14 months of average salary (€1,206.34 (10)) plus 23.75% employer contributions, for 230 workdays/year. |

Table S4: Adjustments and impact determination in the Diabetes Case Study

| Outcomes | Indicators | Deadweight (a) | Attribution (b) | Impact | Rationale |
| --- | --- | --- | --- | --- | --- |
| PATIENTS | | | | | |
| Improved Quality of Life (emotional, physical, and social well-being) | QALYs gained from SMBG | 2.0% | 20.0% | €228,943,263.34 | **(a)** % of registered diabetes patients who used sensors instead of SMBG in 2019.  **(b)** % attributed to diabetes patient support associations. |
| Increased free time | Number of free days gained from reduced time spent in care for non-active patients | 2.0% | 0.0% | €2,471,050.02 | **(a)** % of registered diabetes patients who used sensors instead of SMBG in 2019.  **(b)** Days already estimated based solely on the activity itself; no other compensating factors were identified. |
| FAMILY MEMBERS AND/OR CAREGIVERS | | | | | |
| Improved emotional well-being | Number of family members/caregivers feeling emotionally better | 2.0% | 20.0% | €34,097,734.91 | **(a)** % of registered diabetes patients who used sensors instead of SMBG in 2019.  **(b)** % attributed to diabetes patient support associations. |
| Increased free time | Number of family members/caregiver days saved by not needing to attend medical appointments | 2.0% | 0.0% | €247,105.00 | **(a)** % of registered diabetes patients who used sensors instead of SMBG in 2019.  **(b)** Days already estimated based solely on the activity itself; no other compensating factors were identified. |
| Increased household economic capacity due to reduced need for informal care | Number of diabetes patients avoiding severe complications and no longer needing caregiver | 2.0% | 20.0% | €6,032,989.10 | **(a)** % of registered diabetes patients who used sensors instead of SMBG in 2019.  **(b)** % attributed to diabetes patient support associations. |
| HEALTH SYSTEM / PROVIDERS | | | | | |
| Cost and resource optimization via reduced complication incidence | Number of severe complications avoided | 2.0% | 0.0% | €6,235,009.86 | **(a)** % of registered diabetes patients who used sensors instead of SMBG in 2019.  **(b)** Days already estimated based solely on the activity itself; no other compensating factors were identified. |
|  | Number of patients no longer requiring rehabilitation | 2.0% | 0.0% | €98,167.14 |  |
|  | Number of diabetes patients avoiding dialysis | 2.0% | 0.0% | €16,287,346.45 |  |
| Reduced costs and resources utilization via process optimization | Number of medical visits avoided due to glucose monitoring | 2.0% | 0.0% | €22,021,453.79 |  |
| HEALTHCARE PROFESSIONALS | | | | | |
| Greater job satisfaction | Number of public primary care physicians (GPs) | 2.0% | 30.0% | €573,396.03 | **(a)** % of registered diabetes patients who used sensors instead of SMBG in 2019.  **(b)** % attributed to patient cooperation during follow-up. |
| SOCIETY | | | | | |
| Increased productivity | Number of work absence days avoided due to reduced medical visits among working-aged diabetes patients | 2.0% | 0.0% | €11,131,409.70 | **(a)** % of registered diabetes patients who used sensors instead of SMBG in 2019.  **(b)** Days already estimated based solely on the activity itself; no other compensating factors were identified. |
| Total Impact |  |  |  |  | **€328,138,925.33** |

## Details about HF Case Study

Table S5: Quantities and valuation of outcomes in the HF Case Study

| Outcomes | Indicators | Quantity (a) | Outcome valuation (b) | Value | Rationale |
| --- | --- | --- | --- | --- | --- |
| PATIENTS | | | | | |
| Improved emotional well-being | Number of patients with confirmed HF diagnosis feeling emotionally better | 27,719 | Psychological support | €720.00 | **(a)** Considering that 65.7% (17) of the 42,190 diagnosed HF patients (18) adhered to therapy.  **(b)** One psychology session/month, for one year, considering market prices (€60). |
|  | Number of patients with HF ruled out who feel more at ease | 38,822 | Psychological support | €60.00 | **(a)** Difference between suspected cases (n=81,012) and confirmed diagnoses (n=42,190) (18).  **(b)** One annual psychology session, considering market prices (€60). |
| Improved physical well-being | Number of patients with confirmed HF diagnosis reporting improved physical condition | 27,719 | Physical activity | €228.33 | **(a)** Same 65.7% of diagnosed patients who adhered to therapy.  **(b)** Hydrogymnastic monthly fee of €20.76, considering market prices. Annualized value considering 11 months. |
| Improved social well-being | Number of patients with confirmed HF diagnosis reporting better quality of life and more availability for leisure activities | 27,719 | Average elderly person’s spending on leisure activities | €547.50 | **(a)** Same 65.7% of diagnosed patients who adhered to therapy.  **(b)** Half of average annual leisure spending of an elderly person (€1,095 (4)). |
| Increased free time | Number of additional free days gained by non-working-age patients from avoided medical visits and exams during diagnosis process | 3,731 | Average daily consumption per person | €12.00 | **(a)** 0.5 days (19) per avoided medical appointment (n=5,620) and 0.25 days (19) per avoided echocardiogram (n=21,137) among patients aged 65+, discounting a total of 2,679 days spent performing the test.  **(b)** Average cost of a meal, considering market prices. |
| FAMILY MEMBERS AND/OR CAREGIVERS | | | | | |
| Improved emotional well-being | Number of family members/caregivers feeling emotionally more stable | 27,719 | Psychological support | €240.00 | **(a)** One caregiver per HF patient adhering to therapy. **(b)** Four psychology sessions/year, considering market prices (€60). |
| Increased free time | Number of caregiver days gained from not attending medical appointments/exams | 933 | Average daily consumption per person | €12.00 | **(a)** 25% of the patients’ free time days (n=3,731), assuming 1 in 4 brings a companion.  **(b)** Average cost of a meal, considering market prices. |
| Increased economic capacity of affected families | Number of families no longer needing an active family member as caregiver | 154 | Difference between national minimum wage and average informal caregiver allowance | €3,170.88 | **(a)** 0.56% of patients adhering to therapy (n=27,719).  **(b)** Annual difference between minimum wage (20) and informal caregiver subsidy (=1 social support index (IAS)) (21) in 2019. |
| HEALTH SYSTEM / PROVIDERS | | | | | |
| Resource optimization and cost reduction | Number of avoided medical appointments | 5,620 | NHS follow-up appointment cost | €31.00 | **(a)** Medical appointments avoided with the prescription of B-type natriuretic peptide test in primary care vs. current diagnostic pathway (18).  **(b)** The cost of a follow-up medical appointment according to the NHS price list (8). |
|  | Number of avoided echocardiograms | 21,137 | NHS Echocardiogram cost | €40.70 | **(a)** Echocardiograms avoided with the prescription of B-type natriuretic peptide test in primary care (18).  **(b)** Price of an echocardiogram according to the Publicly Reimbursed Parameters for Primary Care 2021 - code 1530.4 (16). |
|  | Number of avoided hospitalizations | 1,242 | Hospitalization cost due to decompensated HF | €1,430.07 | **(a)** Based on 1,890 avoidable hospitalizations (18), adjusted for 65.7% therapy adherence (17).  **(b)** Average price of DRG 194, severities 1 and 2 (22). |
| Increased treatment costs | Number of patients starting treatment earlier | 25,905 | Average cost of early-phase treatment per patient | €33.52 | **(a)** Early initiation treatment with the prescription of B-type natriuretic peptide test in primary care (18).  **(b)** Average early treatment cost (18). |
| HEALTHCARE PROFESSIONALS | | | | | |
| Greater job satisfaction | Number of general practitioners (GPs) in public primary care centers reporting increased job satisfaction | 5,575 | HF patient management training | €60.00 | **(a)** GPs in primary care.  **(b)** One 4-hour annual training in management of HF patients, according to market values. |
| SOCIETY |  |  |  |  |  |
| Increased productivity | Number of work absence days avoided due to reduced medical visits, exams, and hospitalizations among working-age patients | 3,519 | Average daily cost per worker | €66,63 | **(a)** 0.5 days (19) per medical appointment (n=5,620), 0.25 days (19) per echo (n=21,137), and 9.8 days (19) per hospitalization (n=1,242) in working-age patients (25–64 years, 20.82% (18)), discounting a total of 704 days lost performing the tests.  **(b)** Weighted daily wage based on 14 months of salary (€1,206.34 (10)) plus employer contributions (23,75%), adjusted by weekdays for tests and full calendar for hospitalizations. |

Table S6: Adjustments and impact determination in the HF Case Study

| Outcomes | Indicators | Deadweight (a) | Attribution (b) | Impact | Rationale |
| --- | --- | --- | --- | --- | --- |
| PATIENTS | | | | | |
| Improved Emotional Well-being | Number of patients with confirmed HF diagnosis feeling emotionally better | 38.6% | 10.0% | €11,028,546.33 | **(a)** % of patients starting HF treatment within 1 month via current pathway.  **(b)** % attributed to patient support organizations. |
|  | Number of patients with excluded HF diagnosis who feel more at ease | 32.0% | 40.0% | €950,362.56 | **(a)** % of patients with diagnostic exclusion within 1 month via current pathway.  **(b)** % attributed to echocardiogram use. |
| Improved Physical Well-being | Number of patients with confirmed HF diagnosis who feel physically better | 38.6% | 10.0% | €3,497,400.52 | **(a)** % of patients starting HF treatment within 1 month via current pathway.  **(b)** % attributed to patient support organizations. |
| Improved Social Well-being | Number of patients with confirmed HF diagnosis who experience better quality of life and participate in leisure activities | 38.6% | 10.0% | €8,386,290.44 | **(a)** % of patients starting HF treatment within 1 month via current pathway.  **(b)** % attributed to patient support organizations. |
| Increased Free Time | Number of free days gained by non-working patients from avoided medical visits and exams | 0.0% | 0.0% | €44,767.22 | Days already reflect the direct impact of the activity. No other compensating factors were identified. |
| FAMILY MEMBERS AND/OR CAREGIVERS | | | | | |
| Improved Emotional Well-being | Number of family members/caregivers feeling emotionally more stable | 38.6% | 10.0% | €3,676,182.11 | **(a)** % of patients starting HF treatment within 1 month via current pathway.  **(b)** % attributed to patient support organizations. |
| Increased Free Time | Number of caregiver days gained from not attending medical visits/exams | 0.0% | 0.0% | €11,191.81 | Days already reflect the direct impact of the activity. No other compensating factors were identified. |
| Increased Economic Capacity of Affected Families | Number of families no longer needing an active family member as caregiver | 38.6% | 10.0% | €269,831.77 | **(a)** % of patients starting HF treatment within 1 month via current pathway.  **(b)** % attributed to patient support organizations. |
| HEALTH SYSTEM / PROVIDERS | | | | | |
| Resource Optimization and Cost Reduction | Number of avoided medical appointments | 0.0% | 0.0% | €174,220.00 | Avoided appointments already reflect the direct impact of the activity. No other compensating factors were identified. |
|  | Number of avoided echocardiograms | 0.0% | 0.0% | €860,275.90 | Avoided echos already reflect the direct impact of the activity. No other compensating factors were identified. |
|  | Number of avoided hospitalizations | 0.0% | 0.0% | €1,775,754.61 | Avoided hospitalizations already reflect the direct impact of the activity. No other compensating factors were identified. |
| Increased Treatment Costs | Number of patients starting treatment earlier | 0.0% | 0.0% | –€868,317.00 | Earlier treatments already reflect the direct impact of the activity. No other compensating factors were identified. |
| HEALTHCARE PROFESSIONALS | | | | | |
| Greater Job Satisfaction | Number of general practitioners (GPs) in public primary care centers reporting increased job satisfaction | 0.0% | 50.0% | €167,250.00 | **(a)** Unlikely without the activity.  **(b)** % attributed to existing protocols between hospital and primary care centers. |
| SOCIETY |  |  |  |  |  |
| Increased Productivity | Number of work absence days avoided due to reduced medical visits, exams, and hospitalizations | 0.0% | 0.0% | €234,461.32 | Days already reflect the direct impact of the activity. No other compensating factors were identified. |
| Total Impact |  |  |  |  | **€30,208,217.58** |

## Details about Lung Cancer Case Study

Table S7: Quantities and valuation of outcomes in the Lung Cancer Case Study

| Outcomes | Indicators | Quantity (a) | Outcome valuation (b) | Value | Rationale |
| --- | --- | --- | --- | --- | --- |
| PATIENTS | | | | | |
| Increased survival for patients with stage IV lung adenocarcinoma | Number of patients with increased survival | 692 | Value of 1.45 additional life years for a lung cancer patient | €34,462.44 | **(a)** Patients with stage IV adenocarcinoma that are eligible for targeted therapies.  **(b)** Value of a statistical life year (€158,448 (23)) adjusted by lung cancer disability weight (0.15), i.e., €23,767.20. With 1.45 more years of survival compared to chemotherapy (24), total value is estimated at €34,462.44. |
| Improved emotional well-being | Number of patients who feel more positive due to eligibility for targeted therapy | 692 | Psychological support | €1,440.00 | **(a)** Patients with stage IV adenocarcinoma that are eligible for targeted therapies.  **(b)** Two psychology sessions/month for one year, considering market prices (€60). |
|  | Number of lung cancer patients feeling emotionally better due to avoiding aggressive treatments such as IV chemotherapy | 692 |  |  |  |
| Improved physical well-being | Number of lung cancer patients maintaining physical capacity | 692 | Physical activity | €440.87 | **(a)** Patients with stage IV adenocarcinoma that are eligible for targeted therapies.  **(b)** Gym membership (€36.74/month) for one year. |
|  | Number of lung cancer patients no longer requiring concomitant treatments due to lower toxicity | 692 | Cost of antiemetics and iron supplements | €193.87 | **(a)** Patients with stage IV adenocarcinoma that are eligible for targeted therapies.  **(b)** Common side effects of chemotherapy include anemia; relevant products include ondansetron and iron supplements. Estimated market prices used. |
| Improved social well-being | Number of lung cancer patients feeling able to engage in social and leisure activities | 692 | Annual average leisure spending per adult | €1,260.50 | **(a)** Patients with stage IV adenocarcinoma that are eligible for targeted therapies.  **(b)** Half of average adult annual leisure spending (€2,521.00 (4)). |
| Reduced complications from IV therapy administration | Number of avoided IV-related complication events (e.g., bruising) | 415 | Cost of managing complications | €14.79 | **(a)** 10% of patients receiving IV therapy may experience complications. Based on 6 cycles/patient, estimated 4,155 sessions. **(b)** One unit of thrombocid and oral diclofenac over 18 weeks. |
| Increased free time | Number of non-working patients with more free time due to less time spent in hospital | 432 | Average daily consumption per person | €12.00 | **(a)** Patients with stage IV adenocarcinoma likely to benefit from target therapies (n=692) adjusted to the percentage of cases in non-working age (62.5% (25)).  **(b)** Average cost of a meal, considering market prices. |
| FAMILY MEMBERS AND/OR CAREGIVERS | | | | | |
| Improved emotional well-being | Number of family members/caregivers feeling emotionally more stable | 692 | Psychological support | €1,440.00 | **(a)** One caregiver per patient.  **(b)** Two psychology sessions/month for one year, considering market prices (€60). |
| Improved social well-being | Number of family members/caregivers who maintain social/leisure activities due to patient stability | 692 | Annual average leisure spending per adult | €1,260.50 | **(a)** One caregiver per patient.  **(b)** Half of average adult annual leisure spending (€2,521.00 (4)). |
| Reduced burden of formal/informal care | Number of family members/caregivers no longer needing to provide or hire care | 692 | Annual cost of caregiving to the household | €8,880.00 | **(a)** One caregiver per patient.  **(b)** Average annual household expenditure on informal care, considering market values. |
| HEALTH SYSTEM / PROVIDERS | | | | | |
| Reduced chemotherapy needs | Number of avoided chemotherapy sessions | 4,155 | Cost per chemo session | €496.30 | **(a)** Considering an average of 6 treatment cycles per patient, 4,155 chemotherapy sessions are estimated.  **(b)** DRG cost for outpatient chemotherapy (22). |
| Improved patient safety due to fewer adverse events | Number of patients potentially avoiding chemo-related adverse events | 21 | Annual average cost of adverse events | €92,907.00 | **(a)** Assuming 3% of patients (n=692) experience an adverse event during chemo treatment (26).  **(b)** Estimated cost of cancer-related adverse events (27). |
| Increased treatment costs | Number of lung cancer patients receiving targeted therapy | 692 | Annual cost of targeted therapy per patient | €48,285.72 | **(a)** Patients with stage IV adenocarcinoma eligible for targeted therapies.  **(b)** Market prices for dacomitinib and crizotinib. |
| HEALTHCARE PROFESSIONALS | | | | | |
| Greater job satisfaction | Number of oncologists and pulmonologists reporting greater satisfaction | 348 | Oncology conference participation | €753.56 | **(a)** Assuming that 42.6% (28) of 817 specialists, including oncologists and pulmonologists (29), were dedicated to lung cancer treatment.  **(b)** Average cost of major conferences (ASCO/ESMO). |
| SOCIETY |  |  |  |  |  |
| Increased productivity | Number of working-age lung cancer patients maintaining employment during treatment | 260 | Average annual cost per worker | €20,899.84 | **(a)** Assuming that 37.5% (25) of 692 patients are of working age.  **(b)** 14 months of salary (€1,206.34/month (10)) plus 23.75% employer contributions. |

Table S8: Adjustments and impact determination in the Lung Cancer Case Study

| Outcomes | Indicators | Deadweight (a) | Attribution (b) | Impact | Rationale |
| --- | --- | --- | --- | --- | --- |
| PATIENTS | | | | | |
| Increased survival for patients with stage IV lung adenocarcinoma | Number of patients with increased survival | 0.0% | 0.0% | €23,863,308.44 | The estimated value is already directly attributed to the activity vs. comparator. |
| Improved emotional well-being | Number of patients who feel more positive due to eligibility for targeted therapy | 5.0% | 10.0% | €852,537.00 | **(a)** Not all patients feel more positive.  **(b)** % attributed to cancer patient associations. |
|  | Number of lung cancer patients feeling emotionally better due to avoiding aggressive treatments such as IV chemotherapy |  |  |  |  |
| Improved physical well-being | Number of lung cancer patients maintaining physical capacity | 5.0% | 0.0% | €290,010.59 | **(a)** Some may not experience improvement. **(b)** The estimated value is already directly attributed to the activity vs. comparator. |
|  | Number of lung cancer patients no longer requiring concomitant treatments due to lower toxicity | 5.0% | 0.0% | €127,531.91 | **(a)** Some may still need supportive treatment, due to their overall state of health.  **(b)** The estimated value is already directly attributed to the activity vs. comparator. |
| Improved social well-being | Number of lung cancer patients feeling able to engage in social and leisure activities | 5.0% | 0.0% | €829,184.33 | **(a)** Not all patients feel able.  **(b)** The estimated value is already directly attributed to the activity vs. comparator. |
| Reduced complications from IV therapy administration | Number of avoided IV-related complication events (e.g., bruising) | 10.0% | 0.0% | €5,530.27 | **(a)** Impact also depends on patient status and administration environment.  **(b)** The estimated value is already directly attributed to the activity vs. comparator. |
| Increased free time | Number of non-working patients with more free time due to less time spent in hospital | 5.0% | 0.0% | €4,924.80 | **(a)** Some may not enjoy more free time.  **(b)** The estimated value is already directly attributed to the activity vs. comparator. |
| FAMILY MEMBERS AND/OR CAREGIVERS | | | | | |
| Improved emotional well-being | Number of family members/caregivers feeling emotionally more stable | 5.0% | 10.0% | €852,537.00 | **(a)** Not all family members/ caregivers feel more positive.  **(b)** % attributed to cancer patient associations. |
| Improved social well-being | Number of family members/caregivers who maintain social/leisure activities due to patient stability | 5.0% | 0.0% | €829,184.33 | **(a)** Not all family members/ caregivers feel able.  **(b)** The estimated value is already directly attributed to the activity vs. comparator. |
| Reduced burden of formal/informal care | Number of family members/caregivers no longer needing to provide or hire care | 5.0% | 10.0% | €5,257,311.53 | **(a)** The need for assistance also depends on patient comorbidities and physical status.  **(b)** % attributed to cancer patient associations, for the support they can provide (directly or with agreements at lower prices) in formal or informal care. |
| HEALTH SYSTEM / PROVIDERS | | | | | |
| Reduced chemotherapy needs | Number of avoided chemotherapy sessions | 0.0% | 0.0% | €2,061,959.63 | The estimated value is already directly attributed to the activity vs. comparator. |
| Improved patient safety due to fewer adverse events | Number of patients potentially avoiding chemo-related adverse events | 5.0% | 0.0% | €1,833,487.39 | **(a)** Reducing the occurrence of adverse events also depends on a set of measures adopted by hospitals.  **(b)** The estimated value is already directly attributed to the activity vs. comparator. |
| Increased treatment costs | Number of lung cancer patients receiving targeted therapy | 0.0% | 0.0% | -€33,435,155.2 | The estimated value is already directly attributed to the activity vs. comparator. |
| HEALTHCARE PROFESSIONALS | | | | | |
| Greater job satisfaction | Number of oncologists and pulmonologists reporting greater satisfaction | 21.0% | 10.0% | €186,575.22 | **(a)** Remaining satisfaction from other alternative therapies.  **(b)** % attributed to research institutions. |
| SOCIETY |  |  |  |  |  |
| Increased productivity | Number of working-age lung cancer patients maintaining employment during treatment | 5.0% | 0.0% | €5,162,260.60 | **(a)** The patient’s ability to continue working also depends on their physical condition.  **(b)** The estimated value is already directly attributed to the activity vs. comparator. |
| Total Impact |  |  |  |  | **€9,573,724.87** |

# References

1. López Seguí F, Estrada Cuxart O, Mitjà I Villar O, Hernández Guillamet G, Prat Gil N, Maria Bonet J, et al. A Cost-Benefit Analysis of the COVID-19 Asymptomatic Mass Testing Strategy in the North Metropolitan Area of Barcelona. Int J Environ Res Public Health. 2021 Jun 30;18(13).

2. Instituto Nacional de Estatística. Produto interno bruto (B.1*g) por habitante a preços correntes (Base 2016 - €) por Localização geográfica (NUTS - 2013); Anual [Internet]. INE, Contas económicas regionais. 2021 [cited 2023 Jan 26]. Available from: https://www.ine.pt/xportal/xmain?xpid=INE&xpgid=ine_indicadores&indOcorrCod=0009975&contexto=bd&selTab=tab2

3. Heneghan C, Brassey J, Jefferson T. The Centre for Evidence-Based Medicine. 2020 [cited 2023 Mar 17]. COVID-19: What proportion are asymptomatic? Available from: https://www.cebm.net/covid-19/covid-19-what-proportion-are-asymptomatic/

4. Instituto Nacional de Estatística. Inquérito às Despesas das Famílias: 2015-2016 [Internet]. Lisboa; 2017 [cited 2023 Feb 2]. Available from: https://www.ine.pt/xurl/pub/277098526

5. Mathieu E, Ritchie H, Rodés-Guirao L, Appel C, Gavrilov D, Giattino C, et al. COVID-19 Pandemic [Internet]. OurWorldinData.org. 2020 [cited 2023 Jan 27]. Available from: https://ourworldindata.org/coronavirus

6. Our World in Data. Number of COVID-19 patients in hospital [Internet]. 2024 [cited 2023 Jan 28]. Available from: https://ourworldindata.org/grapher/current-covid-patients-hospital?time=earliest..2021-12-31&country=~PRT

7. Seringa J, Pedreiras S, Freitas MJ, De Matos RV, Rocha J, Millett C, et al. Direct Costs of COVID-19 Inpatient Admissions in a Portuguese Tertiary Care University Centre. Portuguese Journal of Public Health. 2022 Apr 19;40(1):26–34.

8. Diário da República. Portaria n.^o^ 207/2017, de 11 de julho [Internet]. Diário da República n.^o^ 132/2017, Série I de 2017-07-11. 2017 [cited 2023 Feb 20]. Available from: https://diariodarepublica.pt/dr/detalhe/portaria/207-2017-107669157

9. Instituto Nacional de Estatística. Estatísticas da Saúde: 2021 [Internet]. 2023 [cited 2023 Feb 10]. Available from: https://www.ine.pt/xurl/pub/11677508

10. Instituto Nacional de Estatística. Ganho médio mensal (€) por Localização geográfica (NUTS - 2013); Anual. MTSSS/GEP, Quadros de pessoal. 2020.

11. Instituto Nacional de Estatística. Produto interno bruto (B.1*g) a preços correntes (Base 2016 - €) por Localização geográfica; Trimestral . INE, Contas nacionais trimestrais. 2022.

12. Talboom-Kamp E, Ketelaar P, Versluis A. A national program to support self-management for patients with a chronic condition in primary care: A social return on investment analysis. Clinical eHealth. 2021 Jan 1;4:45–9.

13. Vale S, Dores J, Pedro E. PROGRAMA NACIONAL PARA A DIABETES: Desafios e estratégias 2021 [Internet]. Lisboa; 2021 [cited 2022 Dec 27]. Available from: https://www.dgs.pt/programa-nacional-para-a-diabetes/ficheiros-upload/arquivo/relatorio-pnd-2021.

14. Pinto C, Mendes Z. Relevância do Diagnóstico In Vitro na Otimização Clínica e Económica da Gestão da Diabetes. Revista Portuguesa de Diabetes. 2020;15(4):106–13.

15. Sociedade Portuguesa de Diabetologia. Diabetes: Factos e Números – O Ano de 2016, 2017 e 2018 − Relatório Anual do Observatório Nacional da Diabetes. Lisbon; 2019 Dec.

16. Administração Central do Sistema de Saúde IP. Tabela MCDT Convencionados 2021 [Internet]. 2021 [cited 2023 Feb 14]. Available from: https://www.acss.min-saude.pt/2016/10/03/tabelas-meios-complementares-de-diagnostico-e-terapeutica/

17. Catarino P. Conhecimento Sobre a Doença e Comportamentos de Adesão de Doentes com Insuficiência - Dissertação de Mestrado em Gestão e Economia da Saúde Cardíaca. Universidade de Coimbra; 2016.

18. Fonseca C, Bettencourt P, Brito D, Febra H, Pereira Á, Genovez V, et al. NT-proBNP for heart failure diagnosis in Primary Care: Costs or savings? A budget impact study. Revista Portuguesa de Cardiologia. 2022 Mar 1;41(3):183–93.

19. Gouveia MR de A, Ascenção RMS e. S, Fiorentino F, Costa JNMPG da, Broeiro‐Gonçalves PM, Fonseca MCFG da, et al. Current costs of heart failure in Portugal and expected increases due to population aging. Revista Portuguesa de Cardiologia. 2020 Jan 1;39(1):3–11.

20. Diário da República. Decreto-Lei n.^o^ 117/2018, de 27 de dezembro. Diário da República n.^o^ 249/2018, Série I de 2018-12-27. 2018.

21. Diário da República. Portaria n.^o^ 24/2019, de 17 de janeiro. Diário da República n.^o^ 12/2019, Série I de 2019-01-17. 2019.

22. Diário da República. Portaria n^o^ 254/2018, de 7 de setembro [Internet]. Diário da República, 1^a^ série, n^o^ 173. 2018 [cited 2023 Feb 22]. Available from: https://diariodarepublica.pt/dr/detalhe/portaria/254-2018-116353279

23. Schlander M, Schaefer R, Schwarz O. Empirical Studies On The Economic Value Of A Statistical Life Year (VSLY) In Europe: What Do They Tell US? Value in Health. 2017 Oct;20(9):A666.

24. Simarro J, Pérez-Simó G, Mancheño N, Ansotegui E, Muñoz-Núñez CF, Gómez-Codina J, et al. Impact of Molecular Testing Using Next-Generation Sequencing in the Clinical Management of Patients with Non-Small Cell Lung Cancer in a Public Healthcare Hospital. Cancers (Basel). 2023 Mar 10;15.

25. RON. Registo Oncológico Nacional de Todos os Tumores na População Residente em Portugal, em 2019 [Internet]. Instituto Português de Oncologia do Porto, editor. 2022 [cited 2023 Apr 21]. Available from: https://ron.min-saude.pt/pt/biblioteca/publicacoes-ron/ron-2019/

26. Weingart SN, Zhang L, Sweeney M, Hassett M. Chemotherapy medication errors [Internet]. Vol. 19, The Lancet Oncology. 2018. Available from: www.thelancet.com/oncology

27. Ranchon F, Salles G, Späth HM, Schwiertz V, Vantard N, Parat S, et al. Chemotherapeutic errors in hospitalised cancer patients: Attributable damage and extra costs. BMC Cancer. 2011 Nov 8;11.

28. Barata F, Fidalgo P, Figueiredo S, Tonin FS, Duarte-Ramos F. Limitations and perceived delays for diagnosis and staging of lung cancer in Portugal: A nationwide survey analysis. Vol. 16, PLoS ONE. Public Library of Science; 2021.

29. Instituto Nacional de Estatística. Estatísticas da Saúde: 2020 [Internet]. Lisboa; 2022 [cited 2025 May 29]. Available from: www: <url:https://www.ine.pt/xurl/pub/436989156>
